# The relationship between Vitamin D deficiency and polycystic ovary syndrome

**DOI:** 10.4314/ahs.v20i4.45

**Published:** 2020-12

**Authors:** Feyzi Gokosmanoglu, Attila Onmez, Hasan Ergenç

**Affiliations:** 1 Department of Endocrinology, Ordu Medical Park Hospital, Ordu, Turkey; 2 Department of Internal Medicine, Duzce University Medicine Faculty, Düzce, Turkey; 3 Department of Internal Medicine, Sinop Ayancık State Hospital, Sinop, Turkey

**Keywords:** Polycystic ovarian syndrome, vitamin D deficiency, androgen hormones; testosterone

## Abstract

**Background:**

Vitamin D deficiency is frequently seen in patients with polycystic ovary syndrome (PCOS) and has been shown to exhibit multiple effects on the disease process. The purpose of this study was to investigate the role of vitamin D deficiency in complex PCOS pathophysiological pathways.

**Methods:**

Two hundred sixty-seven patients with PCOS were divided into two groups Group 1 with 25(OH)D3 deficiency, and Group 2 with normal 25(OH)D3. Biochemical and hormonal parameters (androgen hormones, gonadotropins, and thyroid function tests) were compared between the two groups.

**Results:**

Eighty-six percent of the patients (n=231) were in Group 1 and 14% (n=36) in Group 2. Statistically significantly higher concentrations of serum testosterone, dehydroepiandrosterone-sulfate and LH were determined in Group 1 (p<0.05). 25(OH)D3 concentrations were negatively correlated with body mass index (r=−0.459), serum testosterone (r =−0.374) and dehydroepiandrosterone-sulfate levels (r=−0.418); (all; p< 0.05).

**Conclusion:**

The study findings show that low 25(OH)D3 levels are associated with high androgen levels in women with PCOS. Vitamin D deficiency should be considered as an additional risk factor in the development of PCOS. We think that providing vitamin D supplementation for women from identified deficiency areas can reduce the risk of PCOS development.

## Introduction

Polycystic ovary syndrome (PCOS) is an endocrine disease frequently seen in women of reproductive age. PCOS is characterized by polycystic ovarian morphology, hyperandrogenism, and ovulatory impairment 1. The etiology of PCOS is still unclear. However, evidence suggests a multi-factorial origin, with expression being seen in women with a genetic disposition 2. The basic finding in the pathophysiology of PCOS is insulin resistance^3^. This develops in association with weight gain and an increase in waist circumferencem and is powerfully associated with hyperandrogenemia and ovarial dysfunction 4. Obesity and insulin resistance aggravate hyperandrogenemia 5. The incidence of cardiovascular diseases, type 2 diabetes mellitus, hypternsion, endometrial cancer, and inflammation-related conditions increases in association with increased adipose tissue and hypernadrtogenemia in women with PCOS 6,7,8.

Vitamin D deficiency also increases the risk of numerous chronic medical conditions such as obesity, cardiovascular disease, type 2 diabetes, malignancy, autoimmunity, infectious disease, and psychological disorder 9. Obesity is a well-recognized risk factor for vitamin D deficiency 10. A negative correlation between body mass index and serum 25(OH)D3 concentrations has been shown in women with PCOS in previous reports^11^. Vitamin D receptor (VDR) is expressed in several tissues other than skeletal muscle. It is therefore associated with glucose metabolism, in addition to the well-known calcium-bone metabolism, the cardiovascular system, malignancies, and female reproductive tissues such as the ovaries, placenta, endometrium, and fallopian tubes^12^.

The relationship between PCOS and vitamin D has already been the subject of various previous studies. Vitamin D deficiency may increase the risk of PCOS^13^. Another study showed that vitamin D support improves blood pressure profiles and reduces insulin resistance, and total testosterone and androstenedione levels in patients with PCOS 14. Lower vitamin D levels have been reported in obese women with PCOS compared to non-obese PCOS patients 15. Impaired glucose tolerance and an increased risk of type 2 diabetes development occur with increasing insulin resistance in PCOS, and an increased risk of type 2 diabetes has been shown in vitamin D deficiency 17. One meta-analysis also reported that the incidence of PCOS increased in vitamin D deficiency, and that this also leads to metabolic and endocrine dysfunction 18.

The prevalence of PCOS is growing. Understanding the mechanism involved may help elucidate the pathophysiology of the syndrome. It is important to identify effective diagnostic and treatment methods in order to better identify the causes of PCOS. The purpose of the present study was to evaluate the association between serum vitamin D deficiency and the risk of disease, and to analyze the relationships with metabolic and endocrine disorders in women with PCOS in our own population.

## Methods

This study involved patients followed-up with diagnosis of PCOS at the Medical Park Hospital endocrinology and gynecology clinics, Ordu, Turkey, between January 2015 and September 2019. Two hundred sixty-seven Caucasian women of reproductive age between 18 and 40 with PCOS were included in the study.

Diagnosis of PCOS was based on the ESHRE/ASRM (Rotterdam) 2004 criteria 19. These consisted of the presence of at least two of ovulation abnormalities such as oligo-ovulation or anovulation, clinical signs of hyperandrogenism and/or biochemical hyperandrogenism, and polycystic ovarian morphology. Patients' ages, anthropometric measurements, Homeostasis Model of Assessment - Insulin Resistance (HOMA-IR) values, lipid profiles, androgen hormones, and thyroid function tests were analyzed.

Exclusion criteria included a history of autoimmune disease, presence of collagen tissue disease, pregnancy, or immunosuppressive medication use, history of diabetes, coronary heart disease, hyperlipidemia, or liver and kidney organ failure, age under 18 or over 40, and menopausal status. Such cases were not included in the study.

All participants underwent physical examination, and their weight, height, and waist circumference (WC) measurements were recorded. Body mass index (BMI) was calculated by dividing weight (in kilograms) by height (in meters) squared. WC measurements were performed at the level of the iliac processes and umbilicus.

Subjects were studied after overnight fasting, and blood specimens were collected at approximately 08:00 hours for the measurement of serum fasting glucose (cut-off level 70–100 mg/dl), fasting insulin (cut-off level 2–5 IU / mL), 25 (OH)D3 (cut-off level 30–50 ng/mL), FSH (cut-off level 1.5 – 12.4 mlU/mL), LH (cut-off level 2.00–15.00 mlU/mL), serum testosterone (cut-off level 3.5–8.6 ng/ml, DHEA-S (cut-off level 82–338 ng/dL), and PRL (cut-off level 2–20 ng/mL) using automated chemiluminescence immunoassay (ICMA) kits (Abbott, IL, USA). Serum 25(OH)D3 levels were used to evaluate vitamin D status. HOMA-IR was calculated using the formula fasting plasma insulin (mg/dL) x fasting plasma glucose (U/mL) / 405.

25 (OH) D3 values were measured using commercial euglobulin clot lysis assay (ECLA) kits (Roche, Germany). Based on Endocrine Society criteria, 25(OH)D3 status was defined as vitamin D deficiency (<20 ng/mL), insufficiency (21–29 ng/mL) or sufficiency (≥ 30 ng/mL) 20. The patients in the present study were divided into two groups - group I: 25(OH)D3 <29 ng/mL and Group II: 25(OH)D3 ≥30 ng/mL.

Pelvic ultrasonography was carried out using a high-resolution apparatus (Philips Affinity 70 ultrasound, Philips North America Corporation 3000 Andover, MA, USA) equipped with a 5-1 MHz broadband convex series probe.

This study was conducted in accordance with the principles enshrined in the Declaration of Helsinki. All participating women gave written informed consent prior to inclusion in the study. The study protocols were approved by the Ordu University Medical Faculty ethical committee.

## Statistical analysis

The study data were analyzed on SPSS version 20.0 software (IBM Corp., USA). Quantitative parametric data were expressed as mean plus standard deviation (SD). The Kolmogorov-Smirnov test was used to analyze the distribution of variables. For non-parametric data, intergroup comparisons were performed using the Mann-Whitney U test, while the independent-t test was used to compare parametric data between the groups. Categorical variables were evaluated using the Pearson Chi-Square Test. Correlations between serum 25(OH) VD3 concentrations, androgen hormones, BMI, waist circumference, HOMA-IR index, thyroid function test, and age were analyzed using Pearson's correlation co-efficient. p values <0.05 were regarded as statistically significant.

## Results

Two hundred sixty-seven patients aged between 18 and 45 (Group 1=231, Group 2=36) were included in the study. Patients' demographic, clinical, biochemical and metabolic characteristics are given in Table 1. All patients were diagnosed with PCOS. Age and triglyceride and LDL-C levels were similar between the two groups. BMI, fasting glucose, HOMA-IR values were statistically significantly higher in Group 1 (25(OH)D3 = 0–29 ng/mL), while Vitamin D, calcium, and HDL-cholesterol levels were higher in Group 2 (25(OH)D3 ≥ 30 ng/mL) (Table 1).

**Table 1:** Patents' clinical, biochemical and metabolic data

Parameters	PCOS		P value
	Group I	Group II	
	25(OH)D_3_ Deficiency (≤29 ng/mL) (n= 231)	25(OH)D_3_ Normal (≥30 ng/mL) (n=36)	
Age(years) mean±SD	28.7±5	29.6±6	0.932
BMI, kg/m^2^	29.1±4.6	25.8±2.4	**0.012**
Waist circumference (cm)	91.5 ± 12.6	82±10.1	**0.031**
Fasting glucose, mg/dl	92.5±12.3	80.1±10.1	**0.025**
HOMA-IR	3.7±1.2	3.3±0.8	**0.037**
25 (OH) D_3._ ng/mL	12.8±3.3	34±7.6	**0.000**
Serum calcium, mg/dL	8.2±2.8	9.6±4.1	**0.029**
Triglyceride, mg/dl	142.8±23.1	136.9±17.6	0.052
LDL-C, mg/dl	143.5±25	144.1±25.2	0.983
HDL-C, mg/dl	40.4±8.8	49.7±9.2	**0.031**

BMI; Body Mass Index. HOMA-IR; Homeostasis Model Assessment Insulin - Resistance Index. LDL-C; LDL-Cholesterol. HDL-C; HDL-Cholesterol. Significant results are shown in bold type.

Serum testosterone, DHEA-S and LH levels were statistically significantly higher in Group 1 (p<0.05). FSH, PRL, TSH, and fT4 values were at similar levels in the two groups, as seen in Table 2. However, PRL and TSH levels were lower in patients with normal Vitamin D levels, although the differences were not statistically significant (p>0.05).

**Table 2:** Comparison of the two groups' hormonal parameters

Parameters	PCOS		*P* value
	Group I	Group II	
	25(OH)D_3_ Deficiency (≤29 ng/mL) (n= 231)	25(OH)D_3_ Normal (≥30 ng/mL) (n=36)	
Serum testosterone, ng/ml	7.4±2.2	5.3±0.9	0.012
DHEA-S, ng/dL	704.8±102.1	501.6±87.8	**0.008**
FSH, mlU/mL	6.8±2.3	6.3±1.5	0.773
LH, mlU/mL	18.5±2.1	9.6±0.7	**0.001**
PRL, ng/mL	23±3.5	15±2.4	0.058
TSH, µIU/L	2.6±0.8	2.4±0.7	0.981
sT4, pmol/L	11.6±2.1	12.2±3	0.936
Serum testosterone, ng/ml	7.4±2.2	5.3±0.9	**0.002**
DHEA-S, ng/dL	704.8±102.1,	501.6±87.8	**0.011**

DHEA-S; Dehydroepiandrosterone Sulfate. FSH; Follicle Stimulating Hormone. LH; *Luteinizing Hormone.* PRL; Prolactin, TSH; Thyroid-Stimulating Hormone, sT4; *Free* Thyroxine. Significant results are shown in bold type.

We observed negative correlation between serum 25(OH)VD3 concentrations and BMI (p=0.002), fasting glucose (p=0.031) waist circumference (p=0.023), HOMA-IR (p=0.006), LH (p=0.027), serum testosterone (p=0.012), and DHEAS (p=0.003) in women with PCOS. No correlation was found between 25(OH)D3 levels and age, FSH, TSH, or sT4 levels in women with PCOS (p>0.05). Correlation analysis is summarized in Table 3.

**Table 3:** Correlation of 25(OH)D_3_ levels and metabolic and endocrine parameters with PCOS

Parameters	*R*	*P*
Age(years)	0.013	0.346
BMI (kg/m^2^)	−0.459	**0.002**
Waist circumference (cm)	−0.315	**0.023**
Fasting glucose, mg/dl	−0.307	**0.031**
HOMA-IR	−0.385	**0.006**
FSH, mlU/mL	−0.134	0.124
LH, mlU/mL	−0.321	**0.027**
Serum testosterone, ng/ml	−0.374	**0.012**
DHEA-S, ng/dL	−0.418	**0.003**
TSH, µIU/L	0.024	0.652
sT4, pmol/L	0.019	0.514

## Discussion

Women diagnosed with PCOS often present with insulin resistance, leading to increased inflammation marker levels, and to a higher risk of type 2 diabetes and cardiovascular disease. These diseases have also been linked to vitamin D deficiency 20. The reason for and nature of this association is still not fully understood. The etiopathogenesis of PCOS is a complex phenomenon arising from the interaction of genetic and environmental factors 21. The results of the present study showed that vitamin D deficiency caused increases in the clinical findings and in the metabolic and hormonal profiles of PCOS patients, which is consistent with other studies 22. The present study also showed that vitamin D deficiency may be a risk factor or may play a role in the pathophysiology of PCOS. There is a powerful association between insulin resistance and PCOS. Vitamin D is known to be one of the factors leading to the development of insulin resistance 23, 24.

An association between PCOS and vitamin D deficiency has been reported in several studies. However, the actual pathogenesis has not yet been elucidated 18. Although the connection between vitamin D deficiency and the underlying causes of PCOS has not yet been clarified, previous studies have revealed a positive correlation between PCOS and body mass index, body fat and insulin resistance^25^, ^26^. Previous studies have also revealed that the changes in intracellular calcium concentrations caused by vitamin D deficiency may lead to ovulation and reproductive abnormalities in PCOS^11^. Statistically significantly higher values for BMI, fasting glucose and HOMA-IR were detected in the vitamin D deficient group in the present research, again in agreement with previous studies (p<0.05). Similarly, a negative correlation was observed between vitamin D levels and BMI, insulin resistance and fasting glucose levels in PCOS patients. The results of the present and previous studies suggest that vitamin D deficiency is a risk factor for PCOS.

PCOS and vitamin D deficiency have been described as risk factors for atherosclerosis and hypertensive disorders. Previous studies have shown that these increase the morbidity and mortality associated with cardiovascular disease 27. Vitamin D replacement has also been shown to reduce systolic blood pressure and mortality associated with cardiovascular disease 18.

However, HDL-C levels were higher in the normal vitamin D group than in the insufficient vitamin D group with PCOS (p<0.05). This finding shows that vitamin D normalization may reduce the cardiovascular disease risk in PCOS patients with vitamin D deficiency. In the literature, as in the present study, vitamin D deficiency has been associated with low HDL cholesterol levels 28,29. Previous studies have also reported that vitamin D deficiency is associated with an imbalance in dehydroepiandrosterone-sulfate, and in hyperandrogenism markers such as serum testosterone, free androgen index, free testosterone, and sex hormone-binding globulin^11^,^22^, ^30^. Serum testosterone, DHEA-S, and LH levels in the present study were higher in Group 1 than in the group with normal vitamin D levels (p<0.05). Our findings show that vitamin D deficiency is linked to a greater increase in androgen hormone levels in PCOS cases. Vitamin D concentrations can serve as a metabolic and hormonal marker in PCOS patients. Replacement therapy with vitamin D has increased insulin sensitivity and reduced androgen levels in PCOS patients with vitamin D deficiency in a number of studies 31.

TSH and PRL levels increased in Group 1, although this was not statistically significant (p>0.05). The National Academy of Clinical Biochemistry has recommended that 2.5 µIU/mL be used rather than 4 µIU/mL for TSH levels 32. We determined a mean TSH level of 2.6 µIU/mL.

Previous studies have shown a connection between PCOS and vitamin D deficiency. There is adequate evidence in the literature to suggest that vitamin D deficiency exacerbates the risk of PCOS 31, 33. The fact that the connection between the two cannot be explained may be due to the complex etiology of PCOS. We think that vitamin D deficiency contributes to PCOS development through insulin resistance, obesity, and an increase in androgen levels. Our findings show that vitamin D deficiency represents a risk factor for PCOS. Individuals living in countries in northern latitudes, with insufficient year-round exposure to sunlight (20–30 min a day), and with insufficient vitamin D intake through diet (fish / fish oil and seafood) require vitamin D supplementation. This will eliminate one factor in the complex etiology of PCOS.
